# The Relation between Intergroup Contact and Subjective Well-Being among College Students at Minzu Universities: The Moderating Role of Social Support

**DOI:** 10.3390/ijerph20043408

**Published:** 2023-02-15

**Authors:** Jian-Hong Ye, Mengmeng Zhang, Xiantong Yang, Mengqin Wang

**Affiliations:** 1 Faculty of Education, Beijing Normal University, Beijing 100875, China; 2 School of Education, Minzu University of China, Beijing 100081, China; 3 Faculty of Psychology, Beijing Normal University, Beijing 100875, China; 4 Department of Lifelong Learning, Simon Fraser University, Vancouver, BC V6B 5K3, Canada

**Keywords:** intergroup contact, social support, subjective well-being, Minzu university, college students

## Abstract

Although Minzu universities provide a platform for communication for college students from all ethnic groups, the multi-ethnic communication pattern could influence students’ well-being. To improve the well-being of these minority college students, this study analyzed the impact of intergroup contact on subjective well-being, as well as the moderating role of social support. Through a cross-sectional investigation, 860 valid data were collected from the Ningxia Hui Autonomous Region. The results found that the quantity of intergroup contact, the quality of intergroup contact, and the global intergroup contact could positively predict the subjective well-being of students at Minzu universities. Social support had a positive moderating effect. That is, the stronger the social support, the stronger prediction it had on subjective well-being from the quantity of intergroup contact, the quality of intergroup contact, and the global intergroup contact among college students at Minzu universities. Therefore, based on the methods of increasing contact opportunities, improving contact quality, and enhancing social support, Minzu universities can increase the interaction among students from all ethnic groups and so, further improve the subjective well-being of college students.

## 1. Introduction

China is a unitary, multi-ethnic state. Because of the diversity of peoples and cultures, the sense of community for the Chinese nation is a policy approach strongly advocated by the government. Therefore, the way of improving communication and integration has become one of the most hotly debated foci for all social sectors/communities in China. It is worth mentioning that Minzu universities (i.e., a university with students of different ethnic groups) provide a platform for communication for college students from all ethnic groups, and have also facilitated exchange, communication, and integration among people [1]. However, the multi-ethnic communication pattern created by a certain university could cause psychological conflicts, as conducting studies away from one’s hometown in a new environment may not be comfortable for students in terms of their values, customs, habits, etc. Therefore, the improvement in students’ well-being is influenced [2,3]. Specifically, college students from various ethnic groups are expected to face the problem of reconstructing interpersonal relationships, as well as adapting to a new living environment and a new study environment. If a higher degree of difference between the new living environment and the previous one is perceived by individuals, they are more likely to feel psychological pressure (i.e., anxiety), which can then influence their level of subjective well-being, and even their mental health [4]. Therefore, starting from the perspective of psychology, this study investigated the situation of intergroup contact of college students at Minzu universities in China, and further explored the relation between intergroup contact and the subjective well-being of individuals, as well as the role played by external supporting resources.

## 2. Literature Review

Improving the positive intergroup contact among college students at Minzu universities is one of the most important aspects of realizing national solidarity. It is also one of the ways of achieving cultural inclusion and emotional closeness between peoples. Nowadays, the analysis of communication and exchange among college students from various ethnic groups has basically remained at the macro-level. Most researchers place the issue of exchange and integration among college students in the context of macro policies, for example, exploring the importance of education policies about national solidarity and development [5,6]. In recent days, academics have emphasized the need to identify the internal psychological factors which have underlying/potential influence on the national awareness/recognition in order to improve national solidarity. It is a transition from psychological self-identity to a homogeneous ideology on a group level. Therefore, it is useful to view this issue from a psychological perspective, and to solve real social issues through empirical investigation. In psychology, the Intergroup Contact Theory starts from the group’s perspective, and discusses the conditions and mechanisms among them. It is considered as an effective strategy to improve intergroup relations and intergroup friendship [7]. In this case, this research utilizes Intergroup Contact Theory as the theoretical ground to analyze the different intergroup contact relations among college students in various Minzu universities.

According to Intergroup Contact Theory, the contact among members from various groups can effectively reduce the intergroup bias. This refers to the inequitable cognitive, emotional, or behavioral response to other groups by valuing or privileging members of one’s own group over other groups and their members by either directly or indirectly devaluing or disadvantaging them [8,9]. This research utilizes this theory to discuss the relation between intergroup contact and the subjective well-being of college students at Minzu universities. Subjective well-being is the assessment of the degree of satisfaction of individuals with life, including the positive emotional status demonstrated by individuals. Subjective well-being, as one of the most important indicators for measuring mental well-being, is influencing the growth and development of ethnic minority students [10]. Nowadays, there are a small number of studies discussing the relation between intergroup contact and well-being. Tip et al. discovered that the contact between ethnic minority groups and mainstream groups can help to improve the subjective well-being of people from ethnic minority groups [11]. The research from Yang et al. found that the contact with individuals from other ethnic groups had a direct positive prediction on subjective well-being, based on the research of the relation between the intergroup contact and subjective well-being among Tibetan students at higher vocational colleges in China [12]. Similarly, research by Laar et al. at a US university found that the sharing of lodgment by students from various racial groups helped to reduce previous bias among groups as time went by [13]. It improved intergroup identity and produced positive emotions. Furthermore, positive emotions are an important part of happiness [14]. Therefore, it is inferred that intergroup contact can reduce stereotypes against ethnic minority students, establish intergroup friendships, and effectively reduce the anxiety and loneliness in the process of integrating into the local learning and life environment, and further improve individuals’ well-being.

Researchers have generally categorized intergroup contact into both the quantity and quality of the contact [15]. However, there are differing opinions on the relation between both the quantity and quality of intergroup contact and well-being. On one hand, there are opinions that the quantity of intergroup contact could improve communication attitudes, and then create well-being. For example, research by Grigoryev et al. shows that more frequent contact with people from other ethnic groups helps to improve the attitude during exchange [16]. The Mere Exposure Theory of Zajonc highlighted that frequent exposure to a new environment allows individuals to produce favorable impressions to others outside of a certain group, because of the contact [17]. There are also opinions that both the quantity and the quality of intergroup contact can improve the attitude of exchange and produce well-being. For example, based on the research of inter-ethnic group friendship among Minzu university students, Chen et al. found that the quantity and quality of intergroup contact could predict the emotions of students from non-local ethnic groups [18]. This finding indicates that the more friends one has from other ethnic groups, and the higher the quality of friendship, the more positive influence there is in terms of the emotions towards people from other ethnic groups, and the higher degree of well-being there is. However, it is the quality rather than the quantity of social contact that has a higher correlation to well-being. For example, in research on the relation between intergroup contact and happiness among migrant elderly people, and among local elderly people, it was found that the quality of intergroup contact significantly predicted the level of happiness of migrant elderly people; however, it could not be predicted by the quantity of intergroup contact [19,20]. Some scholars believe that individual well-being basically comes from establishing a sound interpersonal relationship, rather than the quantity of interpersonal relationships [21]. Only an active social interaction can improve the physical and mental well-being of individuals and reduce the influence of pressure on individuals. Based on the above-mentioned contradictions, it is necessary to analyze the relation between quantity of intergroup contact and well-being, and the relation between quality of intergroup contact and well-being, respectively.

Although the positive contact of college students from various ethnic groups could potentially improve subjective well-being to some extent, if factors of boundary mediation also exist in this mechanism, it could further play the role of prediction of well-being from intergroup contact. Social support refers to the general or specific supporting resources received by individuals from others or from one’s social network [22]. According to various findings, social support has an augmentative function in positive psychology, and positive social support serves as one of the factors in improving the well-being of students [23]. According to the Main Effect Model and the Bumper Model, social support can not only maintain individuals’ positive emotional experience, but it also plays an augmentative role in terms of the sound development of individuals both physically and mentally [24]. High quality social support can provide an emotional echo for ethnic minority college students and improve their recognition of and satisfaction with their lives at universities [25]. On the contrary, when there is a lower degree of accessibility to social support, the negative contact can increase intergroup bias and in turn influence the level of well-being [26]. That is because the Intergroup Contact Theory posits that the contact itself is not enough to reduce bias and decrease intergroup anxiety, only the positive contact that meets the condition can help to form a better intergroup relation and improve well-being [27]. Among them, the external support outside of the group is one of the most important conditions for positive contact, and the interaction between intergroup contact and social support will influence individuals’ subjective well-being. Under various social support conditions, college students with various degrees of intergroup contact present various levels of subjective well-being. Based on this, it is necessary to explore whether social support could moderate the prediction of intergroup contact on subjective well-being. What is more, intergroup contact is the hypernym of the quantity of intergroup contact and of the quality of intergroup contact. It is still unknown whether there is variation in terms of the moderating effect played by social support in the relations between quantity of intergroup contact and well-being, quality of intergroup contact and well-being, and global intergroup contact and well-being. It is therefore necessary to set quantity of intergroup contact, quality of intergroup contact, and global intergroup contact as independent variables, set well-being as a dependent variable, and set social support as a moderator in order to explore the potential differences existing among various variables in terms of the moderating effect.

Exploring the influencing factors of subjective well-being is an effective way to alleviate college students’ mental health issues. This study aimed to investigate the situation of intergroup contact of college students at Minzu universities in China, and further explore the relation between intergroup contact and the subjective well-being of individuals, as well as the role played by external supporting resources. Based on the reviewed literature, the majority of empirical studies have shown that the quantity and quality of intergroup contact have an influence on well-being. Therefore, hypothesis 1 was proposed: the quantity of intergroup contact is positively associated with the well-being of college students at Minzu universities. That is to say, the more intergroup contact there is, the higher level of subjective well-being students will have. Hypothesis 2 was proposed: the quality of intergroup contact is positively associated with the well-being of college students at Minzu universities. That is to say, the higher the quality of intergroup contact, the higher level of subjective well-being students will have.

Additionally, under various social support conditions, college students with various degrees of intergroup contact present various levels of subjective well-being. Hypothesis 3 was proposed: social support could moderate the prediction of intergroup contact on subjective well-being.

## 3. Participants and Methods

This study adopted the cross-sectional method to explore the situation of intergroup contact of college students at Minzu universities in China, and further explored the relation between intergroup contact and the subjective well-being of individuals, as well as the moderating effects of social support. This method can effectively collect large-scale samples, analyze the relationship between variables, and reveal the internal characteristics of students’ psychological development [28]. Subjective well-being is a subjective psychological variable, and quantitative research based on the psychometric method can effectively reveal the relationship between intergroup contact and the well-being of college students in ethnic areas. Thus, this study measured students in the Ningxia Hui Autonomous Region through cluster sampling.

### 3.1. Participants and Procedure

Cluster sampling was adopted. There were 1005 questionnaires sent to the then-enrolled college students at three Minzu universities in the Ningxia Hui Autonomous Region. After invalid responses (i.e., students tended to give consistent answers in the questionnaires) were removed, 860 valid questionnaires were collected, giving an effective recovery rate of 85.57%. Among them, there were 111 males, 749 females; 269 Han students, and 591 students from ethnic minorities (including Hui, Tibetan, Yi, Manchu, Tai, Hani, Lahu, Zhuang, Tujia). The sampling covered college students in every ethnic group, from the first to the fourth year at university. This project was approved by the Ethics Committee of Beijing Normal University (approval number: 202202240017). All students provided their written informed consent. These participants were told that they could voluntarily withdraw at any time, and their responses to the questionnaire would be anonymous and confidential, and that the data collected would be used only in this research.

### 3.2. Measures

#### 3.2.1. Subjective Well-Being

This research adopted the revised version of Campbell’s Index of Well-Being from Li and Zhao [29]. The scale is a students’ self-assessment scale, which can be basically divided into two dimensions—the Index of General Affect and the Satisfaction with Life Index. This scale has nine items and uses a 7-point grading system. Items 1 to 8 explore the Index of General Affect, item 9 explores participants’ satisfaction with life, where the higher the number, the more satisfied they are with their life. The ultimate Index of Happiness was calculated by the sum of the average value of Index of General Affect, along with the Satisfaction with Life Index. The higher the value, the better individuals’ subjective well-being. Previous research also verified that this scale had good internal consistency (Cronbach’s α = 0.93) [30]. In this study, the Cronbach’s alpha was 0.812, which indicated satisfactory reliability.

#### 3.2.2. Intergroup Contact

This research adopted the Intergroup Contact Scale developed by Islam and Hewstone (1993), including two dimensions—the quantity of intergroup contact (two questions) and the quality of intergroup contact (four questions) [15]. The quantity of intergroup contact reflects the number of friends that college students had from other ethnic groups, as well as the number of students from other ethnic groups who had frequent exchange with the participants (e.g., I have many friends from other ethnic groups). The quality of intergroup contact reflects the equality, motivation, relation with members outside a certain community, and satisfaction during the exchange (e.g., I have a pleasant experience during my exchange with students from various ethnic groups). Respondents rated on a 5-point Likert scale. Previous studies indicated that this scale had good internal consistency (Cronbach’s α > 0.7) in China [20]. In this research, the coefficient of internal consistency was 0.892, and the coefficients in secondary scales were 0.840 (quantity of contact) and 0.882 (quality of contact), respectively.

#### 3.2.3. Perceived Social Support

This research adopted the Scale of Perceived Social Support developed by Dahlem et al. to measure the social support felt by students [31]. There are 12 items in this scale, including three dimensions, which are other types of support, family support, and friendship support. This study modified the dimension of “other types of support” to “teacher support”. A 5-point rating was used, which ranged from 1, “strongly disagree,” to 5, “strongly agree.” The higher the score, the more social support the student perceived. Previous studies indicated that this scale had good internal consistency (Cronbach’s α > 0.8) in China [32]. The coefficient of internal consistency in the current study was 0.952.

### 3.3. Data Analysis

After agreement of the leaders (managers) of the universities and of the students themselves, the investigative research was launched on a class-by-class basis, and all questionnaires were collected online. After removing the invalid responses, 860 valid questionnaires were collected. SPSS 23.0 was utilized to perform the descriptive statistical analysis, correlation analysis, and multiple regression analysis.

In addition, to establish the moderating model, this study also explored the moderating effect of social support between intergroup contact and subjective well-being. Specifically, subjective well-being was set as a dependent variable; quantity of intergroup contact, quality of intergroup contact, and global intergroup contact were set as independent variables; social support was set as a moderator; gender, grade, SES, and other variables were set as controlled variables. The analysis was launched to predict the moderating effect of social support in quantity of intergroup contact, in quality of intergroup contact, and in global intergroup contact. In order to avoid collinearity between interaction term and independent variable, and between interaction term and moderator, centralized processing was utilized for the independent variable and moderator.

## 4. Results

### 4.1. Common Method Bias

As this research collected data through the self-reporting of participants, the relation among variables could have been influenced by common method bias. The Harman single factor analysis was adopted to statistically confirm the common method bias [33]. According to the result, before factor rotation, six factors had eigenvalues greater than one. The explained variance for the first factor before rotation was 37.08%, which is lower than the threshold of 40% [34]. Therefore, the influence from the common method bias on data stays within an acceptable range.

### 4.2. Descriptive Statistics and Analysis

The mean, standard deviation, and coefficients of all variables are demonstrated in Table 1. The quantity of intergroup contact was correlated with the quality of intergroup contact, global intergroup contact, well-being, and social support (*r* = 0.649, *p* < 0.01), (*r* = 0.857, *p* < 0.01), (*r* = 0.274, *p* < 0.01), (*r* = 0.635, *p* < 0.01). The quality of intergroup contact was correlated with global intergroup contact, well-being, and social support (*r* = 0.948, *p* < 0.01), (*r* = 0.262, *p* < 0.01), (*r* = 0.687, *p* < 0.01). The global intergroup contact was correlated with well-being and social support (*r* = 0.292, *p* < 0.01), (*r* = 0.230, *p* < 0.01). Well-being was associated with social support (*r* = 0.373, *p* < 0.01).

### 4.3. Prediction of Intergroup Contact on Subjective Well-Being

This research utilized a multilevel regression model to explore the prediction of the quantity and quality of intergroup contact on the well-being of college students (Table 2). In Model 1, the demographic variables of gender, age, and socio-economic status (SES) of the family were included in the first layer of this model. In Model 2a, the quantity of intergroup contact was included in the second layer of the model in order to analyze the prediction of quantity of intergroup contact on well-being. In Model 2b, the quality of intergroup contact was included in the second layer of the model, in order to analyze the prediction of quality of intergroup contact on well-being. Generally speaking, both Model 2a and Model 2b were significant, (*F* (3, 856) = 21.148, *p* < 0.001, *R*^2^ = 0.090; *F* (3, 856) = 19.906, *p* < 0. 001, *R*^2^ = 0.085). The quantity and quality of intergroup contact had a strong prediction on the subjective well-being of college students at Minzu universities (β = 0.413, *t* (856) = 8.245, *p* < 0.001; β = 0.491, *t* (856) = 7.944; *p* < 0.001). This indicates that the more frequent the contact is among students in various ethnic groups, the better the contact is, and there is a higher level of well-being among college students.

### 4.4. Moderating Effect of Social Support

The quantity and quality of intergroup contact positively predicted the subjective well-being of college students at Minzu universities. Furthermore, social support is an important external source of (support) perceived by college students. In this case, we wanted to determine whether the well-being perceived by college students could moderate the relation between quantity of intergroup contact and well-being, the relation between quality of intergroup contact and well-being, and the relation between global intergroup contact and well-being. According to the result, the interaction term of quantity of intergroup contact and social support positively predicted the subjective well-being of college students (β = 0.117, *t* = 2.427, *p* = 0.015), which indicates that social support can directly moderate the relation between intergroup contact and subjective well-being (Table 3). The interaction term of quality of intergroup contact and social support positively predicted the subjective well-being of college students (β = 0.173, *t* = 3.069, *p* = 0.002), which indicates that social support can directly moderate the relation between intergroup contact and subjective well-being (Table 4). The interaction term of global intergroup contact and social support had a strong positive prediction on the subjective well-being of college students (β = 0.175, *t* = 3.110, *p* = 0.002), which indicates that social support can directly moderate the relation between intergroup contact and subjective well-being (Table 5). Making a general comparison of the role played by social support in the relation between quantity of intergroup contact and well-being, the relation between quality of intergroup contact and well-being, and the relation between global intergroup contact and well-being, it was found that the global intergroup contact > quality of intergroup contact > quantity of intergroup contact, which indicates that social support has the strongest prediction in global intergroup contact on well-being.

**Figure 1:**
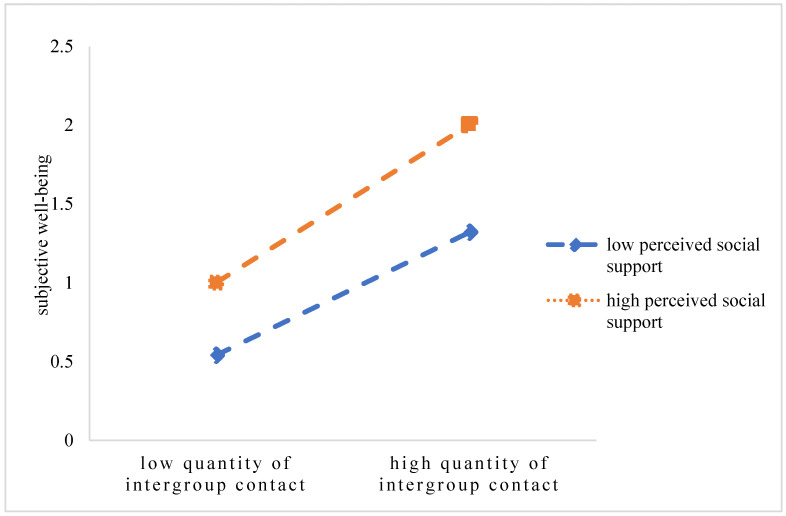
The interaction effects of quantity of intergroup contact and social support on subjective well-being.

**Figure 2:**
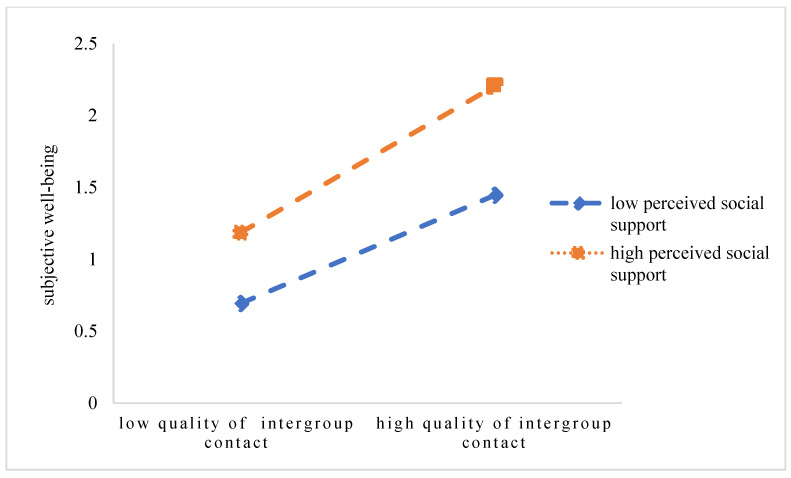
The interaction effects of quality of intergroup contact and social support on subjective well-being.

**Figure 3:**
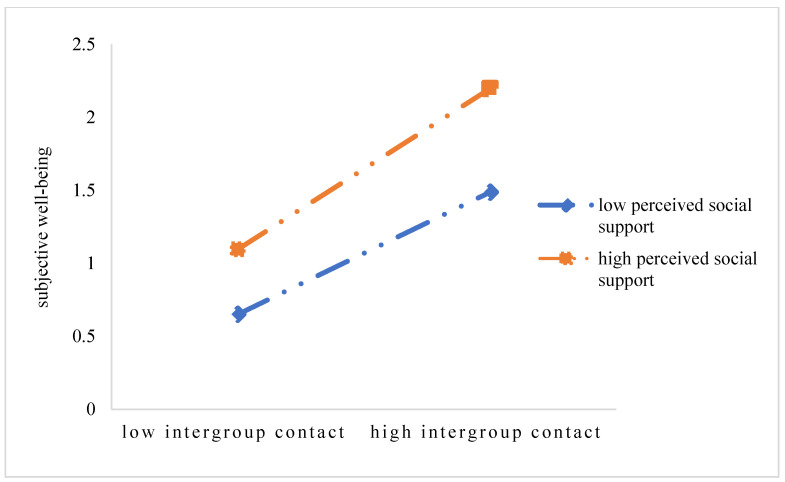
The interaction effects of global intergroup contact and social support on subjective well-being.

In order to further understand the nature of quantity of intergroup contact, quality of intergroup contact, global intergroup contact, and the interaction effect of social support, the figure of interaction effect was drawn with the values of the social support plus and minus one standard deviation, respectively. According to the simple slope test, among the students, as the perceived level of social support rose, all of them—quantity of intergroup contact, quality of intergroup contact, global intergroup contact—had a stronger prediction on subjective well-being. That is to say, higher social support increased the prediction on subjective well-being, from the quantity of intergroup contact, from the quality of intergroup contact, and from global intergroup contact (see Figure 1, Figure 2, and Figure 3, respectively).

## 5. Discussion

### 5.1. Intergroup Contact Has Positive Prediction on Subjective Well-Being

The result of the regression analysis of intergroup contact frequency and subjective well-being confirmed the first hypothesis. Namely, the frequency of intergroup contact of college students at Minzu universities has strong positive prediction on subjective well-being. This result is in accordance with the previous research findings—a higher frequency of intergroup contact can reduce intergroup threat and decrease the negative feelings such as being rejected and being prejudiced against [35]. That is because more frequent contact with students from other ethnic groups and better knowledge of the culture of other ethnic groups can raise the cognition and emotion/affect towards people from other ethnic groups, which effectively reduces the intergroup anxiety and negative feelings [36]. For them, a higher frequency in intergroup exchange and a larger friendship circle with students from other ethnic groups will help to eliminate loneliness and increase well-being.

The result of the regression analysis of the quality of intergroup contact and subjective well-being confirmed the second hypothesis. Namely, the quality of intergroup contact of college students at Minzu universities had a strong positive prediction on subjective well-being. This result is in accordance with the previous research findings—the quality of intergroup contact can effectively predict the well-being of college students [11]. Firstly, the quality of intergroup contact can directly reflect the degree of friendly and positive communication among students from various ethnic groups to some extent. A sound interpersonal relationship is an important source of well-being for individuals. A positive intergroup exchange and interaction helps students from other ethnic groups to gain a more positive emotional experience, and helps to reduce the influence from negative events [37]. Therefore, for college students from Minzu universities, building rapport and establishing sound relationships with students from other ethnic groups helps to enhance their sense of belonging/attachment, and increases their well-being.

### 5.2. Social Support Moderates the Prediction of Intergroup Contact on Subjective Well-Being

According to this research, social support can moderate the relation between quantity of intergroup contact and well-being, the relation between quality of intergroup contact and well-being, and the relation between global intergroup contact and well-being; the above-mentioned results verify the third hypothesis. When there was a higher level of social support, all types of positive intergroup contact (frequency of contact, quality of contact, global intergroup contact) had a positive prediction on the level of well-being of individuals. These research results are in accordance with the theoretical model of social support proposed by Cohen and Wills. Namely, no matter whether it is a positive or stressful experience for individuals, higher level support can increase the sense of control over their environment, improve the degree of satisfaction with university life, and then demonstrate a higher level of well-being [38]. Specifically, for individuals who perceive a higher level of social support, even if there is a lower frequency of group contact, and/or a lower quality of contact, the level of well-being for individuals can still be maintained. That is because, although there are unharmonious factors during the intergroup exchange (i.e., differences in diet among various ethnic groups, differences in customs and habits), the support from significant others can alleviate/buffer the negative influence of intergroup differences. Furthermore, social support provides college students with more positive emotional energy and effective solutions [39]. For example, teachers can guide students to respect the customs and habits of other ethnic groups, and in turn play the role of protection, as well as reduce the negative influence of intergroup contact on their mental health/well-being [40]. Generally, the influence of social support on the subjective well-being of college students at Minzu universities is demonstrated as follows: social support has an augmentative role in subjective well-being; social support allows individuals to gain more positive emotional experiences and stable social relation networks; in the premise of intergroup contact, individuals with a higher degree of social support can demonstrate a higher level of well-being.

It is worth mentioning that compared with the moderating role of social support in the relation between the quantity of intergroup contact and well-being, or in the relation between the quality of intergroup contact and well-being, social support has the most significant moderating effect in the relation between global intergroup contact and well-being. Therefore, among the suggestions and solutions for increasing the well-being of college students, apart from providing high quality social support, it is supposed not only to increase the frequency and quantity of intergroup exchange with students from other ethnic groups, but also to establish a closer relationship with them, and build friendships. Only when combining the effects from the quantity and quality of intergroup contact can social support play its role to the greatest extent, and can have the strongest prediction on the well-being of college students at Minzu universities.

## 6. Limitations and Future Studies

There were several limitations in this research. First and foremost, cross-sectional research is not able to establish the cause-and-effect relation which is applicable to other models. In the coming days, it will be necessary to conduct longitudinal research and experimental investigations to confirm these relations. Secondly, this research only adopted a self-reporting data analysis method, which could have had biased samples, and thus reduce the validity of this research. Therefore, the coming research should adopt various measures to reduce deviation and increase reliability. For example, a qualitative method, third-party observation, experiment, etc., could be adopted. Moreover, qualitative methods (e.g., focus group interviews) can deeply explore the causes of the subjective well-being of college students in ethnic colleges, verify the findings of quantitative studies, and compensate for the drawbacks of one-sidedness of quantitative findings. In addition, this research was completed in a certain region of China, and so the wider application of the research results needs to be further confirmed in other areas. It is essential to adopt an ontological perspective in the study of ethnicity in China. The sense of community for the Chinese nation is a very Chinese and large topic of research. There is currently very little international research on this topic. Therefore, in the future, it should be possible to conduct relevant research from this perspective, which will lead to more research findings based on Chinese characteristics. Finally, in recent years, the government has been promoting the dissemination of good traditional culture and giving people a good sense of cultural confidence. Therefore, in follow-up studies, issues related to traditional culture can also be explored, including innovative curriculum (teaching) design, cultural dissemination, cultural inheritance, etc. 

## 7. Conclusions

This research expands the application of intergroup contact and the mere exposure effect among college students at Minzu universities. Specifically, this research found that the quantity and quality of intergroup contact significantly improved the subjective well-being of college students at Minzu universities, while the social support perceived by college students could effectively moderate the relation between intergroup contact and subjective well-being. That is to say, the higher the social support, the stronger the prediction on subjective well-being from intergroup contact for college students at Minzu universities.

Based on the existing results, this research also makes some practical contributions. Firstly, teachers can provide intergroup contact opportunities and build platforms for students to communicate. Furthermore, universities can create the best possible conditions and improve the quality of contact. The Intergroup Contact Theory holds that the contact itself is not enough to reduce prejudice and anxiety among groups. Only high-quality contact can establish more harmonious intergroup relations. The best practices include building equal status, creating common goals, achieving intergroup cooperation, and providing institutional support [41]. Moreover, enhancing support and cultivating better mental health are also sound solutions. College students at Minzu universities could meet various problems in a new living environment (i.e., anxiety, depression, discomfort, etc.). In solving the problems, extensive and effective social support have significant roles to play [42]. Therefore, building a better social support system for college students at Minzu universities and enhancing their social support are significant measures to improve subjective well-being.

## Figures and Tables

**Table 1:** Descriptive statistics and analysis (*N* = 860).

Variables	Mean	*SD*	1.	2.	3.	4.	5.
1. Quantity of Intergroup Contact	3.872	0.945	1				
2. Quality of Intergroup Contact	4.152	0.767	0.649 ***	1			
3. Global Intergroup Contact	4.059	0.755	0.857 **	0.948 ***	1		
4. Subjective Well-Being	5.086	1.449	0.274 **	0.262 **	0.292 **	1	
5. Perceived Social Support	3.887	0.808	0.635 ***	0.687 ***	0.730 **	0.373 ***	1

Note: *** *p* < 0.001, ** *p* < 0.01.

**Table 2:** Prediction of intergroup contact on subjective well-being of college students at Minzu universities (*N* = 860).

Variables	Subjective Well-Being		
	β in Model 1	β in Model 2a	β in Model 2b
Controlled/Control Variable			
Gender	−0.478	−0.308	−0.461
Grade	−0.218 **	−0.221 **	−0.217 **
SES	0.088 **	0.078 **	0.082 **
Independent Variable			
Quantity of Intergroup Contact		0.413 ***	
Quality of Intergroup Contact			0.491 ***
*R* ^2^	0.018	0.090	0.085
*F*	5.133 **	21.148 ***	19.906 ***
△*R*^2^		0.086	0.081

Note: *** *p* < 0.001, ** *p* < 0.01.

**Table 3:** Analysis of the moderating effect of perceived social support on quantity of intergroup contact and subjective well-being.

Predictor	Subjective Well-Being			
	β	*SE*	*t*	*p*
Quantity of Intergroup Contact	0.413	0.050	8.245	<0.001
Social Support	0.572	0.074	7.725	<0.001
Quantity of Intergroup Contact × Social Support	0.117	0.048	2.427	0.015
*R* ^2^	0.155			
*F*	26.136			
*p*	<0.001			

**Table 4:** Analysis of the moderating effect of perceived social support on the quality of intergroup contact and subjective well-being.

Predictor	Subjective Well-Being			
	β	*SE*	*t*	*p*
Quality of Intergroup Contact	0.491	0.062	7.936	<0.001
Social Support	0.626	0.079	7.949	<0.001
Quality of Intergroup Contact × Social Support	0.173	0.056	3.069	0.002
*R* ^2^	0.156			
*F*	26.269			
*p*	<0.001			

**Table 5:** Analysis of the moderating effect of perceived social support on intergroup contact and subjective well-being.

Predictor	Subjective Well-Being			
	β	*SE*	*t*	*p*
Global Intergroup Contact	0.554	0.062	8.888	<0.001
Social Support	0.578	0.084	6.887	<0.001
Global Intergroup Contact × Social Support	0.175	0.056	3.11	0.002
*R* ^2^	0.158			
*F*	26.603			
*p*	<0.001			

## Data Availability

Data will be made available on request.
